# Using Cell Type–Specific Genes to Identify Cell-Type Transitions Between Different *in vitro* Culture Conditions

**DOI:** 10.3389/fcell.2021.644261

**Published:** 2021-06-25

**Authors:** Xuelin He, Li Liu, Baode Chen, Chao Wu

**Affiliations:** ^1^Department of Nephrology, Beilun People’s Hospital, Ningbo, China; ^2^Kidney Disease Center, The First Affiliated Hospital, Zhejiang University School of Medicine, Hangzhou, China; ^3^Kidney Disease Immunology Laboratory, The Third Grade Laboratory, State Administration of Traditional Chinese Medicine of China, Hangzhou, China; ^4^Department of Library, The First Affiliated Hospital, Zhejiang University School of Medicine, Hangzhou, China; ^5^Department of Laboratory Medicine, The First Affiliated Hospital, Zhejiang University School of Medicine, Hangzhou, China; ^6^State Key Laboratory for Diagnosis and Treatment of Infectious Diseases, National Clinical Research Center for Infectious Diseases, The First Affiliated Hospital, Zhejiang University School of Medicine, Hangzhou, China; ^7^State Key Laboratory for Diagnosis and Treatment of Infectious Diseases, Collaborative Innovation Center for Diagnosis and Treatment of Infectious Diseases, The First Affiliated Hospital, Zhejiang University School of Medicine, Hangzhou, China

**Keywords:** single-cell RNA-seq, bulk RNA-seq, cell type-specific genes, cell-identity, *in vitro* cell culture

## Abstract

*In vitro* differentiation or expansion of stem and progenitor cells under chemical stimulation or genetic manipulation is used for understanding the molecular mechanisms of cell differentiation and self-renewal. However, concerns around the cell identity of *in vitro*–cultured cells exist. Bioinformatics methods, which rely heavily on signatures of cell types, have been developed to estimate cell types in bulk samples. The Tabula Muris Senis project provides an important basis for the comprehensive identification of signatures for different cell types. Here, we identified 46 cell type–specific (CTS) gene clusters for 83 mouse cell types. We conducted Gene Ontology term enrichment analysis on the gene clusters and revealed the specific functions of the relevant cell types. Next, we proposed a simple method, named CTSFinder, to identify different cell types between bulk RNA-Seq samples using the 46 CTS gene clusters. We applied CTSFinder on bulk RNA-Seq data from 17 organs and from developing mouse liver over different stages. We successfully identified the specific cell types between organs and captured the dynamics of different cell types during liver development. We applied CTSFinder with bulk RNA-Seq data from a growth factor–induced neural progenitor cell culture system and identified the dynamics of brain immune cells and nonimmune cells during the long-time cell culture. We also applied CTSFinder with bulk RNA-Seq data from reprogramming induced pluripotent stem cells and identified the stage when those cells were massively induced. Finally, we applied CTSFinder with bulk RNA-Seq data from *in vivo* and *in vitro* developing mouse retina and captured the dynamics of different cell types in the two development systems. The CTS gene clusters and CTSFinder method could thus serve as promising toolkits for assessing the cell identity of *in vitro* culture systems.

## Introduction

Single-cell RNA sequencing (scRNA-Seq) is a powerful tool that can be used to profile gene expression in individual cells (Tang et al., 2009). The 10x Genomics platform and Smart-Seq2 platform provide commercially available scRNA-Seq solutions for researchers worldwide. Massive sequencing of single cells from multiple tissues of model animals, such as that performed during the Tabula Muris Senis project and the Mouse Cell Atlas project, provides characterizations of cells in their respective tissues and have significantly improved our understanding of the transcriptomes of individual cell types, especially those that were previously poorly characterized (Han X. et al., 2018; Tabula Muris Consortium et al., 2018, 2020; Han et al., 2020). These comprehensive scRNA-Seq resources provide an unprecedented opportunity to study unique gene expression programs in different cell types and find cell type–specific (CTS) genes. These genes will enhance our understanding of the specific functions of the cell types and serve as ideal markers of cell identity.

*In vitro* cell culture is a standard tool for understanding cell molecular mechanisms under chemical stimulation, as well as genetic manipulation. It is also an efficient tool for producing stem cells on a large-scale for therapeutic interventions (Polanco et al., 2020). However, concerns around the cell identity of cultured cells exist. The cultured cells, especially stem and progenitor cells, may differentiate and change their identity over time. Immunohistochemistry and flow cytometry have been applied to determining the cell identity of culture cells (Polanco et al., 2020; Shibamiya et al., 2020). However, these traditional methods rely on limited molecular markers and lack scalability relative to the current rate of data generation (Hu et al., 2016). Direct scRNA-Seq is a powerful tool for the identification of cell types in the culture pool. However, the cost of scRNA-Seq is 30 times more than that of bulk RNA-Seq, which thus impedes its diffusion and use.

In recent years, several bioinformatics methods have been developed to estimate cell types in bulk samples from gene expression profiles (Le et al., 2020). These methods, including CIBERSORTx (Newman et al., 2019), Tsoucas et al. (2019), Bisque (Jew et al., 2020), Scaden (Deng et al., 2019), MuSiC (Wang et al., 2019), xCell (Aran et al., 2017), and CPM (Frishberg et al., 2019), rely on the CTS genes to decompose gene expression profiles of bulk samples and estimate cell types in the samples. Using bulk RNA sequencing experiments and these methods, researchers can derive the cell types present in bulk samples. In some circumstances, researchers are more concerned about the different cell types between bulk samples, such as emerging cell populations during *in vitro* cell culture. Gene set enrichment analyses, such as CTen (Shoemaker et al., 2012), ssGSEA (Barbie et al., 2009), and many other methods, provide solutions for identifying the different cell types between bulk samples. However, all these methods rely heavily on CTS genes to estimate cell types.

In most methods, such as CIBERSORTx and xCell, CTS genes are defined as the genes specifically expressed in a unique cell type. Sun et al. (2015); Vrba and Futscher (2018), and other researchers used genes specifically expressed in a limited number of tissues as plasma DNA methylation markers for noninvasive prenatal, cancer, or transplantation assessments. Their findings suggested that genes specifically expressed in a limited number of cell types could also serve as CTS genes. Panina et al. (2020) summarized the CTS gene databases for mice and humans, including Labome, CellFinder (Stachelscheid et al., 2014), CellMarker (Zhang et al., 2019), PanglaoDB (Franzén et al., 2019), and SHOGoiN (Hatano et al., 2011). Multiple cell-type markers collected from heterogeneous experimental sources are available for a cell type in the databases. A major concern is that we need to evaluate the cell-type markers from different sources to understand the scope and limitations before combining them as a marker set for a cell type. However, evaluation of the markers set for a cell type is lacking in the databases.

Here, we identified 46 CTS gene clusters related to 83 mouse cell types using the scRNA-Seq data of more than 350,000 cells from the Tabula Muris Senis project. Gene Ontology (GO) term enrichment analysis of the CTS gene clusters revealed the specific functions of the relevant cell types. We further proposed a simple method named CTSFinder to identify different cell types between bulk RNA-Seq samples based on the 46 CTS gene clusters. We tested CTSFinder with bulk RNA-Seq data from 17 organs and successfully identified the specific cell types of the organs. We further tested CTSFinder with bulk RNA-Seq data from developing mouse liver over different stages and captured the dynamics of different cell types during development. Then, we applied CTSFinder on the bulk RNA-Seq data from a growth factor–induced neural progenitor cells (giNPCs) culture system. We identified the dynamics of brain immune cells and nonimmune cells during the long-time cell culture. We also applied CTSFinder with the bulk RNA-Seq data from reprogramming induced pluripotent stem (iPS) cells by a tamoxifen-inducible Cre recombinase (mER-Cre-mER)–induced Sox2, Klf4, and c-Myc (SKM) expressing system. We identified the stage when those cells were massively induced. Finally, we applied CTSFinder with bulk RNA-Seq data from *in vivo* and *in vitro* developing mouse retina. We identified the shared and unique cell types between the two systems, suggesting the development track of each system. Overall, we identified 46 CTS gene clusters and demonstrated that they could be used to identify the different cell types between mouse bulk RNA-Seq samples.

## Results

### Identification of Mouse CTS Gene Clusters With A Single-Cell RNA-Seq Data Compendium From Tabula Muris Senis

**FIGURE 1:**
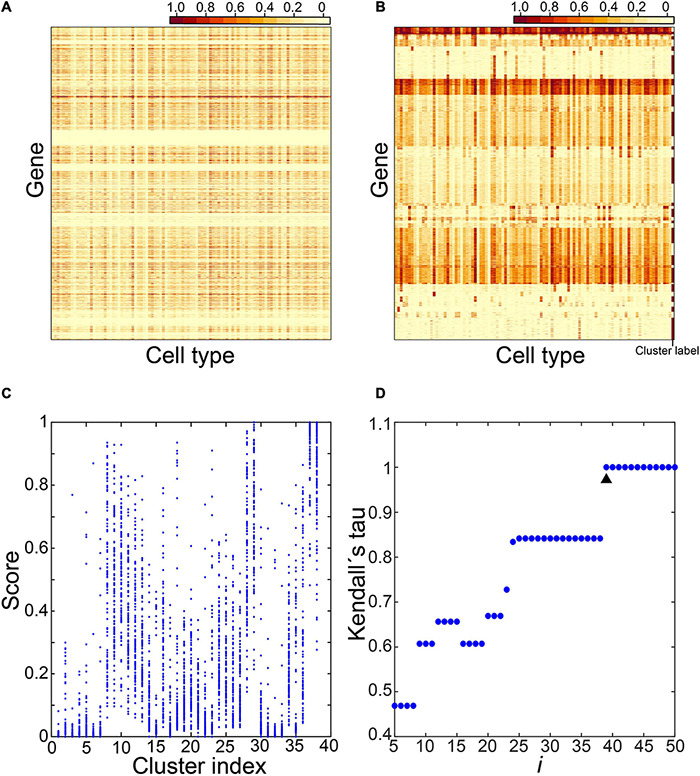
Identification of cell type–specific (CTS) gene clusters. Five steps were involved in identifying CTS gene clusters. The expression values of genes across the 101 cell types in step 1 **(A)**, the expression heatmap of the gene clusters over the 101 cell types in step 2 **(B)**, the expression scores of the gene clusters over the 101 cell types in step 3 **(C)**, and the Kendall rank correlation coefficient between gene clusters under different cluster parameters **(D)** were displayed.

We selected cells from the Tabula Muris Senis project (see “Data” in “Materials and Methods” section), including cells from 3-, 18-, and 24-months-old mice sequenced by a SMART-Seq2 platform; and cells from 1-, 3-, 18-, 21-, 24-, and 30-months-old mice sequenced by a 10x Genomics platform. We grouped cells into cell types by annotation information for each age group. We selected cell types with 20 or more cells and calculated gene expression profiles of the cell types (see “Calculation of Gene Expression Profiles of Cell Types” in “Materials and Methods” section). Thus, we obtained gene expression profiles of cell types in each age group of mice sequenced by either platform (Supplementary Table 1). In the 3-months-old mice sequenced by the SMART-Seq2 platform, we found that most cell types (101) were profiled. We identified CTS gene clusters with the gene expression profiles of these cell types. We took the gene expression profiles of cell types from the other age groups of mice sequenced by either platform to validate the identified CTS gene clusters. We identified the CTS gene clusters with the following steps (Figure 1).

In step 1, we selected candidate genes.

We constructed a gene expression matrix of 22,966 genes in the 101 cell types. Each column represents a cell type and each row a gene (Figure 1A). For each gene, we checked expression values in the 101 cell types and counted the number of cell types with an expression value >0.5 as *h*. We selected 12,823 genes satisfying 1≤*h*≤10.

In step 2, we clustered candidate genes.

We clustered candidate genes by their expression profiles in the 101 cell types. We employed the R package “factoextra” to cluster genes (Kassambara and Mundt, 2019). We used the “euclidean” method to measure the distance between observations followed by the “ward.D2” method to agglomerate the observations. Next, the “fviz_dend” function was used to produce dendrograms; the tree was cut into *i* clusters using the “cutree” function (Figure 1B, here *i* = 38).

In step 3, we calculated expression scores of the gene clusters and the similarity between them.

We selected a gene cluster *s* from the *i* clusters (1≤*s*≤*i*). This cluster included *m* genes. We calculated the expression score of gene cluster *s* in cell type *n* (1≤*n* ≤101) as follows:

$${\text{Score}}_{s_{n}}=\text{Median}\left({\text{exp}}_{1n},{\text{exp}}_{2n},\ldots ,{\text{exp}}_{mn}\right).$$

Here exp_*mn*_ is the expression value of the *m*th gene of gene cluster *s* in cell type *n*. We calculated the expression scores of gene cluster *s* in all 101 cell types.

We calculated the expression scores of all *i* clusters via this method. In Figure 1C, we took*i* as 38 and calculated expression scores of the 38 clusters in the 101 cell types.

Then, for each cluster, we checked the expression scores in the 101 cell types and labeled the cell types with an expression score > 0.5 as 1, and the cell types with an expression score ≤ 0.5 as 0. We randomly selected two clusters, *x* and *y*, and calculated the Kendall rank correlation coefficient between their labeled values (Ken_*xy*_). We calculated the similarity between every two clusters via this method. We identified the maximum value of the Kendall rank correlation coefficients as Ken_max.

In step 4, we determined the optimal number of clusters.

We enumerated *i* from 5 to 50. For each *i*, we repeated steps 2 and 3 to obtain Ken_max_*i*_. We plotted Ken_max_*i*_ under different *i* (Figure 1D). We identified the *i* with Ken_max_*i*_ = 1 and selected the minimum value of them as *i*_min. Finally, we determined the optimal number of clusters as (*i*_min−1) and repeated step 2 to obtain gene clusters.

The choice of *i* determines expression patterns of the resultant gene clusters. A small *i* may produce large gene clusters with genes of various expression levels in a cell type, which cannot help us find gene clusters with clear expression patterns. A large *i* can produce small gene clusters with clear expression patterns. However, it may generate multiple gene clusters sharing the same expression patterns, causing inconvenience in finding all the CTS genes associated with the cell types. We transformed the expression patterns of the resultant gene clusters under each *i* into a binary space with expression score > 0.5 or ≤0.5. The analysis based on the maximum value of Kendall rank correlation coefficients can help us obtain gene clusters with unique expression patterns as many as possible.

In step 5, we identified CTS gene clusters.

We calculated expression scores in the 101 cell types for each gene cluster identified in step 4 via the method described in step 3. Then we checked expression scores in the 101 cell types for each gene cluster and marked the cell types with an expression score > 0.2 as expressed cell types (E types). Those with an expression score > 0.5 were denoted as specific cell types (S types). We counted S type and E type for each gene cluster. Finally, we classified gene clusters into three types: (1) housekeeping gene clusters, with E-type number > 10; (2) CTS gene clusters, with E-type number ≤ 10 and S-type number > 0; (3) undetermined gene clusters, with E-type number ≤ 10, and S-type number = 0.

**FIGURE 2:**
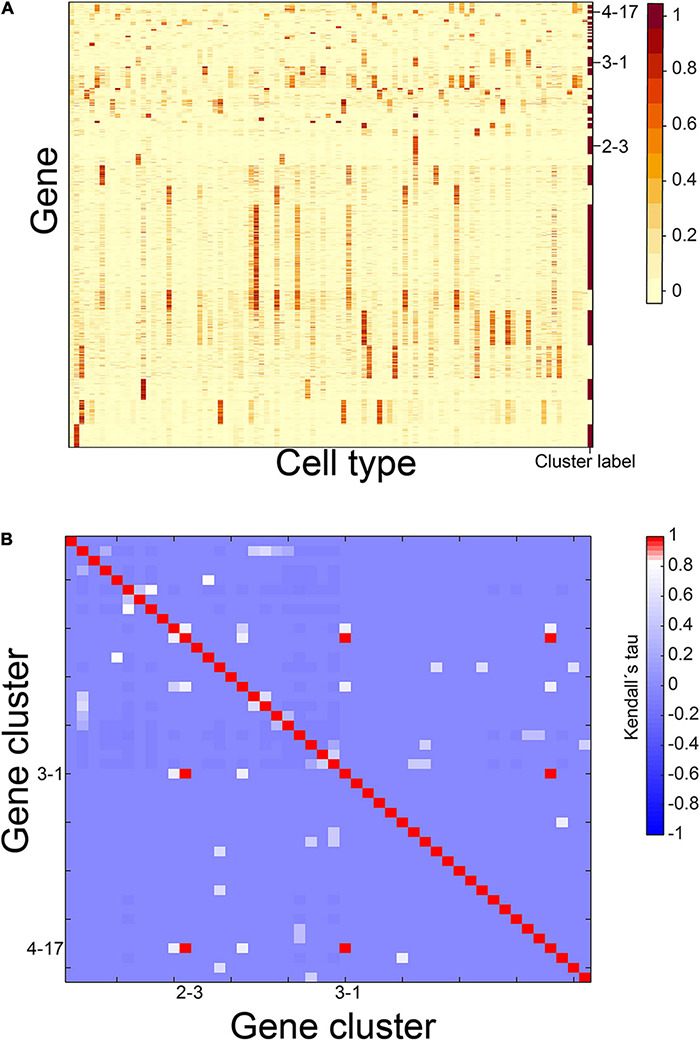
Gene expression patterns of identified CTS gene clusters. **(A)** Expression heatmap of the 46 identified CTS gene clusters. **(B)** Heatmap of Kendall rank correlation coefficients between CTS gene cluster pairs. Genes in the heatmap were sorted by the gene clusters, and the “cluster label” distinguished the genes from different gene clusters.

At first, we conducted the above steps 1–5 to obtain 87 housekeeping gene clusters, nine CTS gene clusters, and five undetermined gene clusters (Supplementary Table 2). We then selected the 1,785 genes in the undetermined gene clusters as candidate genes and ran steps 2–5 above to obtain two housekeeping gene clusters, 15 CTS gene clusters, and seven undetermined gene clusters (Supplementary Table 2). Next, we selected 729 genes in the undetermined gene clusters as candidate genes and ran steps 2–5 above to obtain one housekeeping gene cluster, four CTS gene clusters, and six undetermined gene clusters (Supplementary Table 2). Four hundred eighty-seven genes were in the undermined gene clusters and used as candidate genes to run steps 2–5 again. We obtained one housekeeping gene cluster, 18 CTS gene clusters, and two undetermined gene clusters (Supplementary Table 2). Only 80 genes were in the undermined gene clusters. We stopped here. In total, we identified 46 CTS gene clusters (Supplementary Table 3). Their S types included 61 cell types, and their E types contained 83 cell types (Supplementary Table 4). We calculated expression scores of these gene clusters in 101 cell types (Figure 2A). For each cluster, we labeled the cell types with an expression score > 0.5 as 1, and the cell types with an expression score ≤ 0.5 as 0. We selected all bivariate cluster pairs and calculated Kendall rank correlation coefficients between the labeled values of them. Out of the 2,070 gene cluster pairs, three pairs had coefficients equal to one, involving three gene clusters (Figures 2A,B). We thought we had successfully identified the gene clusters with unique S-type patterns to this end.

### Evaluation of the 46 CTS Gene Clusters

**FIGURE 3:**
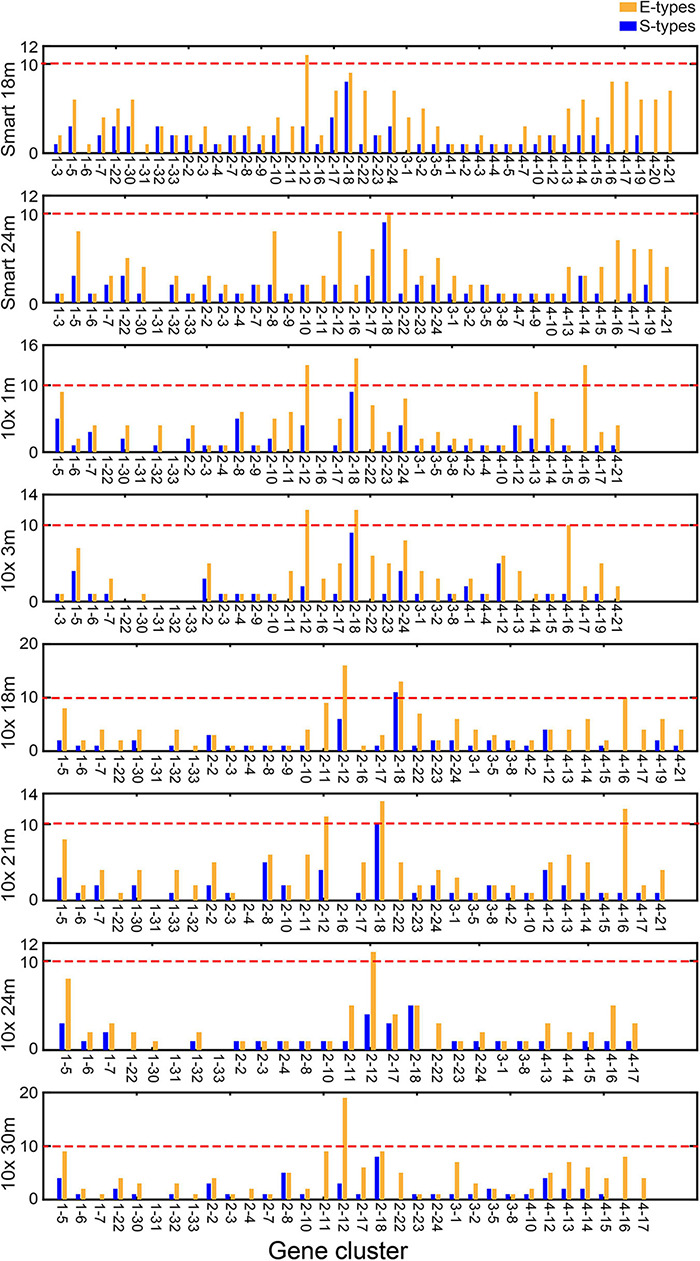
Number of S types and E types associated with each CTS gene cluster in the validated the single-cell RNA sequencing (scRNA-Seq) data. “Smart 18m” and “Smart 24m” represent the scRNA-Seq data using the SMART-Seq2 platform in 18- and 24-months-old mice. “10x 1m,” “10x 3m,” “10x 18m,” “10x 21m,” “10x 24m,” and “10x 30m” represent the scRNA-Seq data using the 10x Genomics platform in 1-, 3-, 18-, 21-, 24-, and 30-months-old mice.

We took the gene expression profiles of cell types from the SMART-Seq2 platform in 18- and 24-months-old mice and the 10x Genomics platform in 1-, 3-, 18-, 21-, 24-, and 30-months-old mice as validated datasets. We calculated expression scores of the CTS gene clusters and then counted E type and S type of each CTS gene cluster in each dataset (Figure 3). We found that 42 CTS gene clusters were validated as CTS gene clusters in one or more dataset(s). Gene clusters 2–12, 2–18, 4–16, and 1–31 failed to be validated as CTS gene clusters in all datasets. We found gene clusters 2–12, 2–18, and 4–16 had more than 10 E types in some datasets (Supplementary Table 5). Gene clusters 2–18 and 4–16 were expressed in types of T cells, and gene cluster 2–12 showed a broad expression in immune cells. The three gene clusters were specifically expressed in types of immune cells. We retained them in the CTS gene cluster list for distinguishing immune cells from other cells. Only gene cluster 1–31 had no S type in all the validated datasets (Figure 3). We found that medium spiny neurons were the S type of gene cluster 1–31 in the test dataset (cells sequenced by the SMART-Seq2 platform in 3-months-old mice). The medium spiny neurons were not sequenced in any validated datasets. We kept gene cluster 1–31 as signatures related to medium spiny neurons. Thus, we retained all the 46 CTS gene clusters as signatures related to specific cell type(s).

**FIGURE 4:**
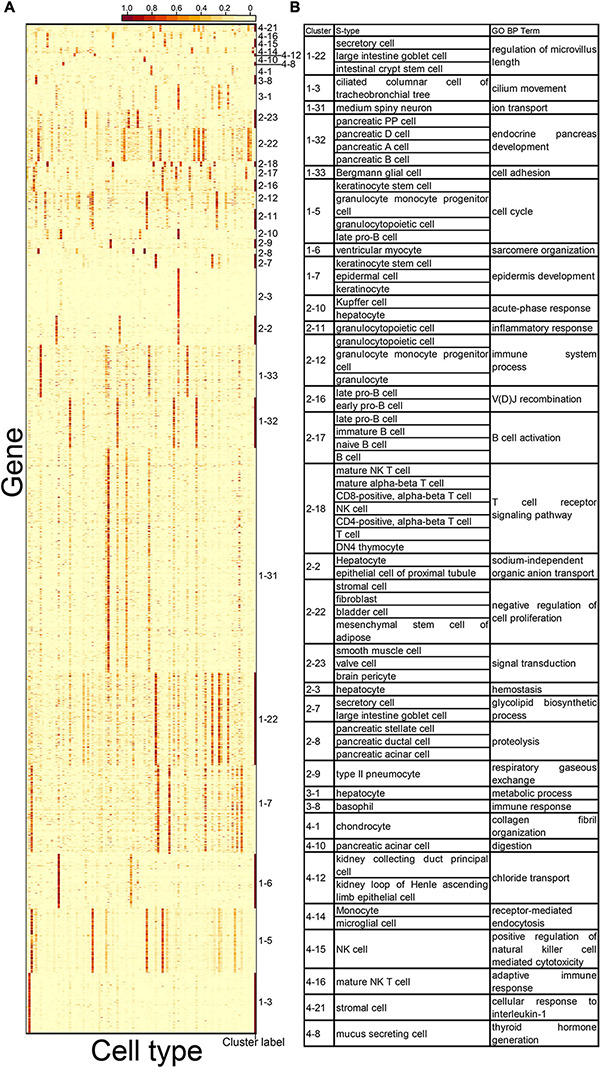
Cell types and gene ontology (GO) terms associated with 31 CTS gene clusters. **(A)** Expression heatmap of 31 CTS gene clusters with enriched GO terms over the 101 cell types. Genes in the heatmap were sorted by the gene clusters, and the “cluster label” distinguished the genes from different gene clusters. The names of the 101 cell types are listed in Supplementary Table 1 (“Smart_3m” column) in the same order. **(B)** S types and selected GO terms of the 31 CTS gene clusters.

**FIGURE 5:**
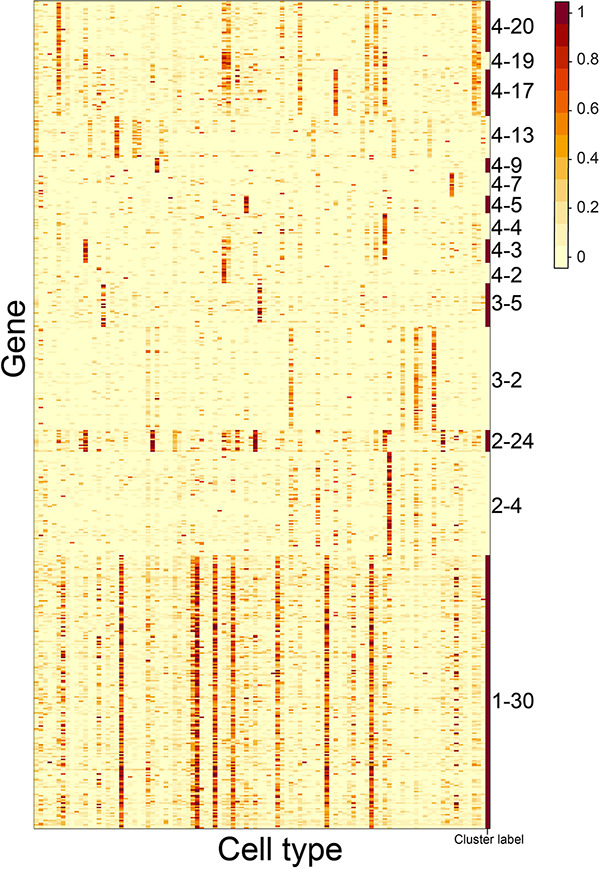
Gene expression patterns of 15 CTS gene clusters without enriched GO terms over the 101 cell types. Genes in the heatmap were sorted by the gene clusters, and the “cluster label” distinguished the genes from different gene clusters. The names of the 101 cell types are listed in Supplementary Table 1 (“Smart_3m” column) in the same order.

Next, we explored the potential functions of the CTS gene clusters. We conducted GO term enrichment analysis on the gene clusters (see “Gene Set Enrichment Analysis” in “Materials and Methods” section). Thirty-one of the 46 gene clusters (67.4%) had enriched GO terms (Figure 4A and Supplementary Table 6), whereas 15 did not (Figure 5). For the 31 gene clusters, we listed their S type(s) and found the enriched terms supported the specific functions of the cell types (Figure 4B). For example, gene cluster 1–3 were specifically expressed in the ciliated columnar cells of tracheobronchial tree tissue; the genes were enriched in the “cilium movement” term. Gene cluster 1–32 was specifically expressed in pancreatic PP cells, pancreatic D cells, pancreatic A cells, and pancreatic B cells; the genes were enriched in the “endocrine pancreas development” term. Gene cluster 2–9 was specifically expressed in type 2 pneumocyte; the genes were enriched in the “respiratory gaseous exchange” term.

**FIGURE 6:**
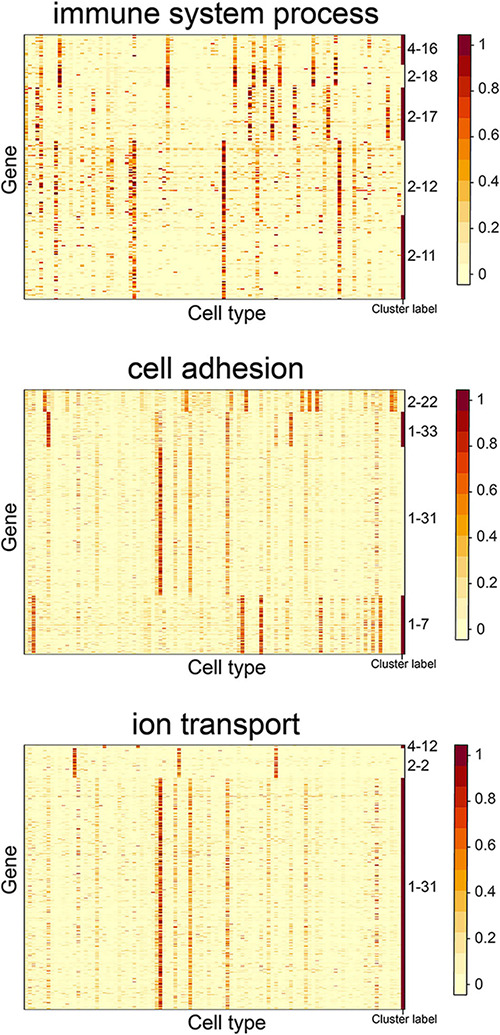
Expression heatmap of the CTS gene clusters enriched in the GO terms “immune system process,” “cell adhesion,” and “ion transport.” Genes in the heatmap were sorted by the gene clusters, and the “cluster label” distinguished the genes from different gene clusters. The names of 101 cell types are listed in Supplementary Table 1 (“Smart_3m” column) in the same order.

We observed that some gene clusters had the same GO term. For example, gene clusters 2–11, 2–12, 2–17, 2–18, and 4–16 were enriched in the “immune system process” term. We examined the heatmap of genes comprising the gene clusters and found they had distinct expression patterns (Figure 6). The S-type profiles showed that gene clusters 2–11 and 2–12 were both specifically expressed in granulocytopoietic cells; gene cluster 2–17 showed specific expression in late pro–B cells, immature B cells, naive B cells, and B cells; gene clusters 2–18 and 4–16 were both specifically expressed in mature natural killer T cells (Supplementary Table 4). The results suggested that the term “immune system process” could be further divided to reflect the processes occurring in different cell types. We also found that the terms “cell adhesion” and “ion transport” could be further divided (Figure 6).

**FIGURE 7:**
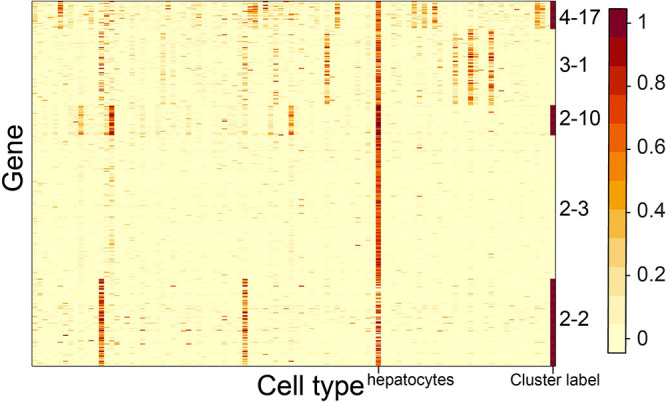
Expression heatmap of the CTS gene clusters specifically expressed in hepatocytes. Genes in the heatmap were sorted by the gene clusters, and the “cluster label” distinguished the genes from different gene clusters.

We observed that multiple CTS gene clusters were specifically expressed in the same cell type. For example, gene clusters 2–2, 2–3, 2–10, 3–1, and 4–17 shared hepatocytes as their S type (Figure 7). The S type and GO term results of gene cluster 2–2 showed that hepatocytes and epithelial cells of proximal tubule tissue both participated in the process of sodium-independent organic anion transport (Figure 4B). Gene cluster 2–3 revealed the unique roles that hepatocytes played in hemostasis (Figure 4B). The S type and GO term results of gene cluster 1–10 showed that hepatocytes and Kupffer cells took part in the process of acute-phase response. These results revealed the multiple functions of hepatocytes, as well as the functional similarity between hepatocytes and other cell types.

Accordingly, we reasoned that CTS gene clusters were applicable across scRNA-Seq datasets and provided rich information on the specific functions of different cell types.

### Identification of Specific Cell Types From Simulated Bulk RNA-Seq Data

We want to determine whether the CTS gene clusters could be used to identify different cell types between bulk samples. We simulated the bulk RNA-Seq data of cells from each of the 101 cell types (see “Construction of the Simulated Datasets” in “Materials and Methods” section for details). We also constructed the simulated bulk RNA-Seq data of the cells from 101 cell types (see “Construction of the Simulated Datasets” in “Materials and Methods” section). We developed a permutation-based method, named CTSFinder, to identify the significant CTS gene sets between bulk samples (see “Permutation-Based Fold Change Test” in “Materials and Methods” section).

**FIGURE 8:**
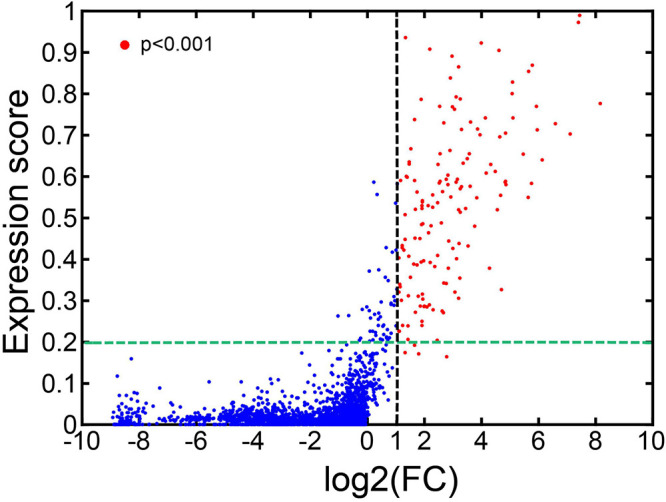
Expression scores and log2(*FC*) values of the CTS gene clusters in 101 cell types.

For each of the 101 cell types, we took their bulk RNA-Seq data as the case and the bulk RNA-Seq data from the cells from all 101 cell types as the control. We ran CTSFinder to calculate the log2 transformed fold change (*log*⁡2(*FC*)) values and *p* values of CTS gene clusters in each cell type. We also calculated the expression scores of the CTS gene clusters in each cell type. We plotted the expression score and *log*⁡2(*FC*) value pairs for CTS gene clusters from the 101 cell types (Figure 8). We identified the significantly up-regulated CTS gene clusters with *log*⁡2(*FC*) > 1 and *p* < 0.001. We found 154 CTS gene clusters were significantly up-regulated, and 150 of them had expression scores greater than 0.2 (Figure 8). The results suggested that the E-type profiles of significant CTS gene clusters could help identify the cell types.

### Identification of Specific Cell Types Between Different Organs From Bulk RNA-Seq Data

We have demonstrated that the CTS gene clusters can help identify the specific cell types in simulated data. We then tested the performance of CTSFinder on bulk RNA-Seq data between different organs. Bulk RNA-Seq profiles from 17 organs from two female and four male, C57BL/6JN, 3-months-old mice were obtained from the outputs of the Tabula Muris Senis project. The 17 organs include bone (both femurs and tibiae), brain (hemibrain), brown adipose tissue (BAT, interscapular depot), gonadal adipose tissue (GAT, inguinal depot), heart, kidney, limb muscle (tibialis anterior), liver, lung, marrow, mesenteric adipose tissue (MAT), pancreas, skin, small intestine (duodenum), spleen, subcutaneous adipose tissue (SCAT, posterior depot), and white blood cells (buffy coat). We found that cells from 14 of the 17 organs had been profiled using a SMART-Seq2 platform in 3-months-old mice. Besides, the large intestine tissue had been profiled with SMART-Seq2 platform in 3-months-old mice. We paired the bulk RNA-Seq data from the small intestine and scRNA-Seq data from the large intestine. Thus, we had both bulk RNA-Seq data and scRNA-Seq data for 15 organs including the brain, BAT, GAT, heart, kidney, limb muscle, liver, lung, marrow, MAT, pancreas, skin, intestine (small or large intestine), spleen, and SCAT.

We took each of the 15 organs as cases in turn, with the combined samples from the other organs as the control. We ran CTSFinder and identified the significantly up-regulated gene clusters for each organ (see “Permutation-Based Fold Change Test” in “Materials and Methods” section). We identified 33 up-regulated gene cluster–organ pairs (Supplementary Table 7). We listed the cell types detected by scRNA-Seq in each organ. Then, for each pair, we matched the E type(s) of the gene cluster and the cell types in the organ. In 31 pairs, the E type(s) of the gene cluster matched the cell types present in the organ (Supplementary Table 7). In two pairs, the E types of gene clusters did not match the cell types present in the organ, namely, gene cluster 1–6 detected in limb muscle and gene cluster 2–24 detected in MAT. It is unexpected to see that 1–6 is up-regulated in limb muscle because its E types, ventricular myocytes, and atrial myocytes are not associated with the production of limb muscle. However, the GO term result of gene cluster 1–6 showed the genes took part in the processes of “sarcomere organization” and “muscle contraction” (Supplementary Table 6). The gene cluster may thus share signatures with a cell type in limb muscle, which had not been profiled by the scRNA-Seq experiment but plays similar roles to ventricular myocytes and atrial myocytes in limb muscle. Gene cluster 2–24, whose E types include multiple cell types (Supplementary Table 7), was significantly up-regulated in MAT. We found no GO terms enriched in the genes. A further Kyoto Encyclopedia of Genes and Genomes (KEGG) enrichment analysis showed that the “vascular smooth muscle contraction” pathway was enriched in the genes (see “Gene Set Enrichment Analysis” in “Materials and Methods” section). The enriched pathway matched the specific functions of some of its E types, smooth muscle cells, and smooth muscle cells of the trachea. However, the roles of the gene cluster in MAT warrant further investigation.

Here we identified 33 significantly up-regulated gene cluster–organ pairs, and 32 of them could be explained. The results thus demonstrated that we could identify specific cell types in organs by analyzing CTS gene cluster expression from bulk RNA-Seq data.

### Identification of Specific Cell Types Between Different Development Stages From Developing Mouse Liver Bulk RNA-Seq Data

We tested the performance of CTS gene clusters on time-series bulk RNA-Seq data to reveal the dynamics of specific cell types. Renaud et al. used a bulk RNA sequencing experiment to interrogate the developmental dynamics of the C57BL/6 mouse liver transcriptome (Renaud et al., 2014). They profiled the developing mouse liver over 12 different time points from the late embryonic stage (E17.5) to maturity (60 days after birth). Gong et al. used a bulk RNA sequencing experiment to profile developing C57BL/6 mouse liver at 15 different time points that covered embryonic days (E12.5, E13.5, E14.5, E15.5, E16.5, E17.5, and E18.5), postnatal days (D1, D3, and D5), and postnatal weeks (W1, W2, W3, W6, and W8) (Gong et al., 2020). We obtained gene expression profiles at time points E17.5, D0, D1, D3, D5, D10, D15, D20, D25, D30, D45, and D60 from Renaud et al.’s data and gene expression profiles at time points E17.5, E18.5, D1, D3, D5, W1, W2, W3, W6, and W8 from Gong et al.’s data.

We took the data from E17.5 as the control and the data at other time points as the case. We ran CTSFinder and identified the significantly up- or down-regulated gene clusters for each time point in either Renaud et al.’s data or Gong et al.’s data (see “Permutation-Based Fold Change Test” in “Materials and Methods” section). Gene clusters 2–10, 2–2, 2–3, 3–1, 4–17, 2–11, 2–12, 2–16, 2–17, 1–5, 2–23, 2–24, 1–33, and 2–4 were profiled by the two datasets and significantly up- or down-regulated in at least one time point. The E types of gene cluster 2–10 include hepatocytes, Kupffer cells, and endothelial cells of hepatic sinusoid tissue (Supplementary Table 4). The GO term enrichment analysis showed that the genes played roles in the process of “acute-phase response” but not immune-related processes (Supplementary Table 6). The E types of 2–2, 2–3, 3–1, and 4–17 include hepatocytes. We inferred that the five gene clusters were signatures of hepatocytes. The E types of gene clusters 2–11 and 2–12 include cell types related to granulocytes and monocytes. We inferred that the two gene clusters were signatures of granulocyte- and monocyte-related cells. The E types of gene clusters 2–16 and 2–17 include cell types related to B cells. We inferred that the two gene clusters were signatures of B-cell–related cells. The E types of 1–5 are stem and progenitor cells. The GO term enrichment analysis showed that the genes were highly enriched in proliferation-related processes (Supplementary Table 6). We inferred that the gene cluster was a signature associated with stem/progenitor cells in the liver. The E types of gene clusters 2–23 and 2–24 include smooth muscle cells. We conducted KEGG enrichment analysis on the two gene clusters and found both gene clusters were enriched in the “vascular smooth muscle contraction” pathway (see “Gene Set Enrichment Analysis” in “Materials and Methods” section). We inferred that the two gene clusters were signatures of vascular smooth muscle cells in the liver. The E types of gene cluster 1–33 are Bergmann glial cells, astrocytes, oligodendrocyte precursor cells, and neuronal stem cells. The GO term enrichment analysis showed that the genes participated in the process of cell adhesion (Supplementary Table 6). It has been reported that hepatic stellate cells (HSCs) and astrocytes share striking morphological and functional similarities (Schachtrup et al., 2011). The gene cluster could serve as signatures related to HSCs. The E type of gene cluster 2–4 is bladder urothelial cells. We did not find any GO terms enriched in the gene cluster. However, KEGG pathway enrichment analysis showed that the “metabolism of xenobiotics by cytochrome P450” pathway was enriched in the gene cluster (see “Gene Set Enrichment Analysis” in “Materials and Methods” section). The cell type(s) associated with gene cluster 2–4 in the liver needs further investigation.

**FIGURE 9:**
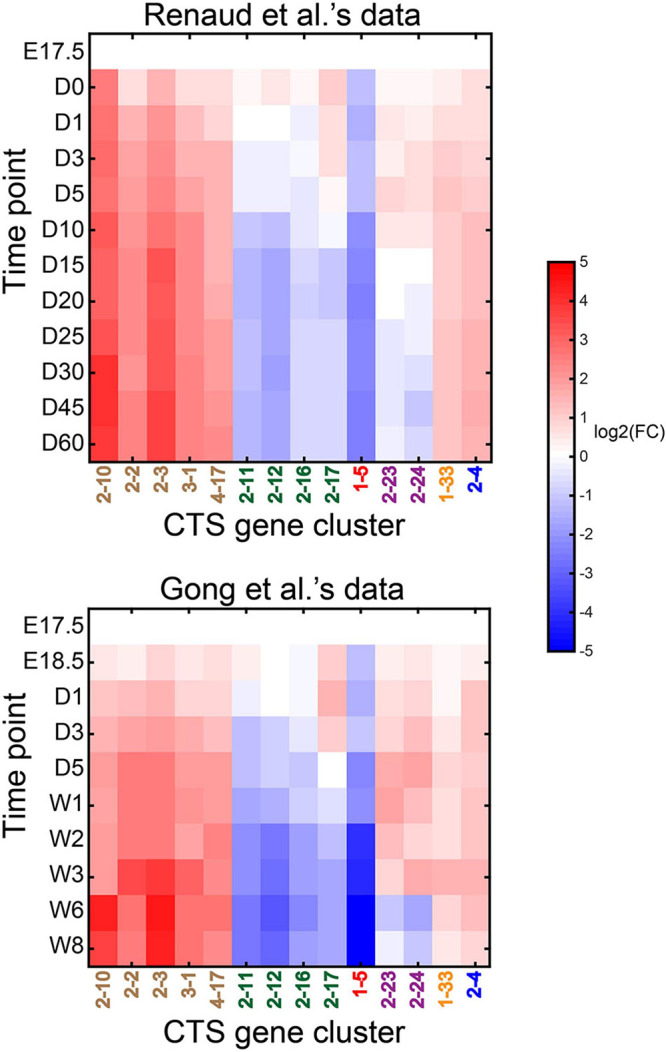
Dynamics of significantly dysregulated CTS gene clusters during mouse liver development. The heatmap displays the expression fold change of the gene clusters during mouse liver development compared to E17.5 time point. The gene clusters in brown font are associated with hepatocytes; those in green are associated with immune cells; the one in red is associated with stem/progenitor cells; those in purple are possibly associated with vascular smooth muscle cells in the liver tissue; the one in yellow is possibly associated with hepatic stellate cells (HSCs). The representative cell type of gene cluster 1–4, in blue, is not determined.

When taking E17.5 as the starting point, the gene clusters associated with hepatocytes (2–10, 2–2, 2–3, 3–1, and 4–17) were up-regulated during the development (Figure 9). The gene clusters associated with granulocytes (2–11 and 2–12) were down-regulated. The gene clusters related to B cells (2–16 and 2–17) were down-regulated. The gene cluster of stem/progenitor cells (1–5) was down-regulated. The gene clusters associated with vascular smooth muscle cells (2–23 and 2–24) were up-regulated from E17.5 to weeks 2 or 3 after birth and then down-regulated. The gene cluster of HSC (1–33) was up-regulated during the development. Gene cluster 2–4 was also up-regulated during development. In summary, the results from two independent datasets were highly consistent.

Gong et al. used proteomics data to reveal five temporal expression modules during mouse liver development from E12.5 to week 8 (Gong et al., 2020). Module 1, mainly involved in cell cycle and RNA transcription, was down-regulated during the development. Module 2, participating in inflammatory response, phagocytosis, and immune response, obtained a peak intensity at E18.5 and then was subsequently down-regulated. Modules 3–5 were enriched in similar biological processes, including oxidation–reduction, metabolism, and transport, which are all essential for adult liver function. They were up-regulated after birth compared to time point E17.5. The results from proteomics data suggested that the time-series intensity profiles of module 1 reflected the dynamics of stem/progenitor cells in the development. The intensity profiles of module 2 reflected the dynamics of immune cells, including granulocytes and B cells, in the development. The time-series profiles of modules 3–5 generally reflected the dynamics of hepatocytes. The dynamics of cell types derived from the bulk RNA-Seq data using the CTS gene clusters were consistent with the dynamics of the cell types derived from proteomics data. We captured the dynamics of different cell types during mouse liver development with the CTS gene clusters.

We used CIBERSORTx to estimate cell fractions in the developing mouse liver bulk RNA-Seq data and compared the cell fractions between different time points (see “Application of CIBERSORTx to Estimate Cell Fractions in Bulk Samples” in “Materials and Methods” section). We identified the cell types with fold change > 2 or fold change <0.5 at any time point and listed them in Supplementary Figure 1. The results revealed that hepatocytes were expanded, and professional antigen-presenting cells, late pro–B cells, granulocytes, and hematopoietic stem cells were reduced during the development process in both datasets. The CTSFinder also captured the dynamics of these cell types in both datasets: gene clusters 2–10, 2–2, 2–3, 3–1, and 4–17 for hepatocytes, 2–11, 2–12, 2–16, and 2–17 for late pro–B cells and granulocytes, and 1–5 for hematopoietic stem cells (Figure 9). However, CTSFinder provided ambiguous results. The results from CIBERSORTx also revealed that many cell types with small cell fractions were expanded or reduced during the development process in only one dataset (Supplementary Figure 1). They needed to be further investigated. However, the gene clusters reported by CTSFinder were highly consistent between the datasets. Besides the cell types revealed by CIBERSORTx, CTSFinder possibly captured the dynamics of vascular smooth muscle cells and HSCs in both datasets, providing more details about mouse liver development.

### Identification of Specific Cell Types Between *in vitro*–Cultured Cells From Bulk RNA-Seq Data

We used CTS gene clusters to identify cell-identity transitions during *in vitro* cell culture. Gao et al. (2017) developed a method to generate giNPCs from mouse embryonic fibroblasts (MEFs). First, they cultured MEFs in an initiation medium for 14 days with the following supplements: B27 minus vitamin A, heparin, leukemia inhibitory factor, basic fibroblast growth factor (bFGF), and epidermal growth factor (EGF). They gently pipetted the cells every day for the first week to prevent them from attaching to the bottom of the dish. Sphere morphology was visualized during the process. Then, the neural rosettes were pipetted and passaged in suspension onto ultralow attachment plates (Costar) to form the giNPCs in the second week. Sphere-like colonies attached to the bottom of the culture dishes, followed by cell mixtures migrating and gradually forming monolayer structures in that week. Next, they digested the cell mixtures and expanded the cells with the following supplements: N2, B27, bFGF, and EGF. This facilitated the establishment of primary neurosphere-like networks in the third week. They harvested cultured cells at various induction days, specifically D1, D4, D7, D10, D14, D17, and D21, and conducted bulk RNA sequencing experiments at each time point. They found that the cultured cells could be divided into three stages: initiation, intermediate, and maturation. At the initiation stage, MEFs were induced by the initiation medium, and a sphere morphology was observed within the first week (D1, D4, and D7). At the intermediate stage, the monolayer structure appeared, and cells started to express NPC-specific genes (D10 and D14). At the maturation stage, primary neurosphere-like networks formed, and NPC-specific genes were prominently up-regulated (D17 and D21).

We took the data from D1 as the control and the data from other time points as the case. We ran CTSFinder and identified the significantly up-regulated gene clusters for each time point (see “Permutation-Based Fold Change Test” in “Materials and Methods” section). Gene clusters 1–30, 1–31, 1–33, 1–5, 2–11, 2–12, 3–5, 4–13, and 4–14 were significantly up-regulated in at least one time point. The E types of 1–30 are medium spiny neurons, neurons, oligodendrocyte precursor cells, neuronal stem cells, Bergmann glial cells, pancreatic D cells, pancreatic A cells, pancreatic B cells, and pancreatic PP cells (Supplementary Table 4). The E types of 1–31 are medium spiny neurons, neurons, and oligodendrocyte precursor cells. The E types of 1–33 are Bergmann glial cells, astrocytes, oligodendrocyte precursor cells, and neuronal stem cells. We inferred that the three gene clusters were signatures associated with brain nonimmune cells. We inferred 1–5 to be signature of stem/progenitor cells. 2–11 and 2–12 were inferred to be signatures of granulocytes and monocytes related cells. The E types of 4–13 and 4–14 include cell types related to monocytes (Supplementary Table 4). The E types of gene cluster 3–5 are endothelial cells of hepatic sinusoid tissue and Kupffer cells. We inferred that 3–5 was signature related to Kupffer cells in the brain tissue. Here, we inferred that 2–11, 2–12, 3–5, 4–13, and 4–14 were the signatures associated with brain immune cells.

**FIGURE 10:**
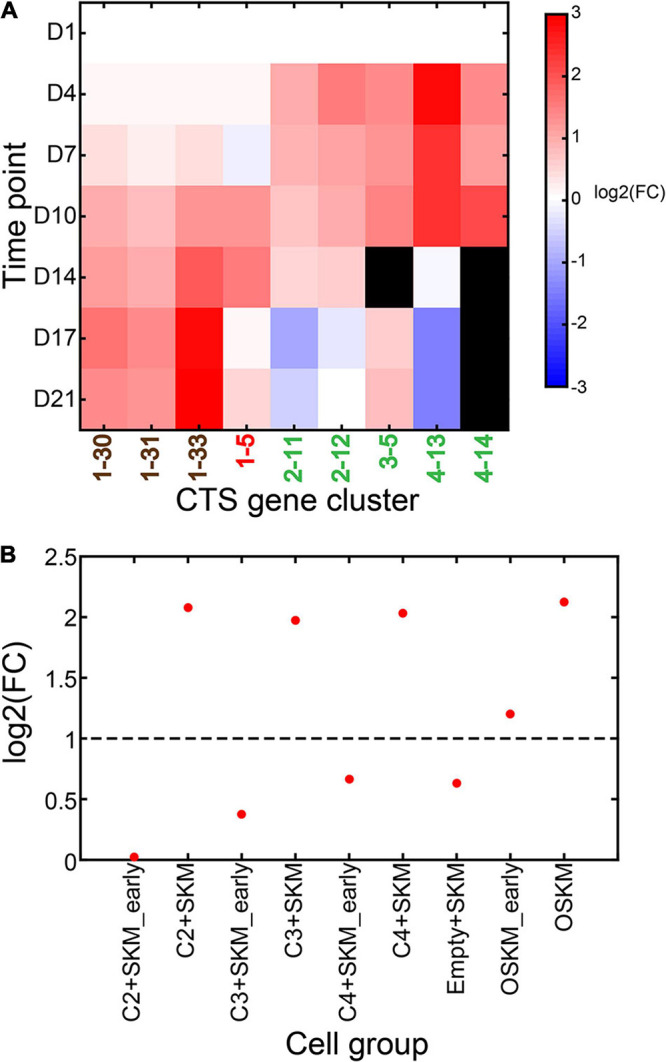
Dynamics of significantly up-regulated CTS gene clusters during the culture of growth factor-induced neural progenitor cells (giNPCs) and induced pluripotent stem (iPS) cells. **(A)** Expression fold change of the significantly up-regulated gene clusters during the culture of giNPCs compared to mouse embryonic fibroblasts (MEFs). The gene clusters in brown font are associated with brain nonimmune cells; the one in red is associated with stem/progenitor cells; those in green are associated with brain immune cells. **(B)** Expression fold change of CTS gene cluster 1–5 under different conditions compared to MEFs.

When taking D1 as the starting point, the gene clusters associated with brain nonimmune cells were up-regulated gradually over the course of 21 days (Figure 10A). The gene set of stem/progenitor cells was up-regulated between D10 and D14 and down-regulated in the third week. The gene clusters related to brain immune cells (2–11, 2–12, 3–5, 4–13, and 4–14) were up-regulated between D4 and D14 and down-regulated during the third week. This suggested that the brain nonimmune cells were gradually differentiating and expanding in the initial, intermediate, and maturation stages. The stem/progenitor cells (giNPCs) were mainly induced in the intermediate stage. The brain immune cells were differentiating during the initial and intermediate stages. The MEFs tended to differentiate into immune cells ahead of nonimmune cells in the initial stage. The manipulation of pipetting and passaging neural rosettes in the intermediate stage facilitated giNPC generation and the differentiation of brain nonimmune cells. The manipulation of digesting the cell mixtures and supplying an expanded medium stimulated giNPCs to differentiate into brain nonimmune cells in the maturation stage to a massive extent.

We also used CIBERSORTx to estimate cell fractions in the cultured giNPCs bulk RNA-Seq data and compared the cell fractions between different time points (see “Application of CIBERSORTx to Estimate Cell Fractions in Bulk Samples” in “Materials and Methods” section). We identified the cell types with fold change > 2 at any time point and listed them in Supplementary Figure 2. The result showed that Kupffer cells, leukocytes, classical monocytes, and monocytes were expanded from D4 to D10 and then reduced. Bergmann glial cells, neuronal stem cells, oligodendrocyte precursor cells, and astrocytes were expanded from D10 to D21. CIBERSORTx revealed the dynamics of these brain immune cells and nonimmune cells in a clear, unambiguous way comparing to CTSFinder. The results from CIBERSORTx also reported other cell types (Supplementary Figure 2), which needed to be further investigated.

Eguchi et al. (2016) conducted genetic manipulation on MEFs using an mER-Cre-mER system. They constructed a genome-scale ATF library and tested it in reprogramming MEF to iPS cells. They found that three combinations of ATFs could induce pluripotency when expressed with SKM, including (1) C2-Zfatf1, Zfatf2, and Zfatf3; (2) C3-Zfatf1, Zfatf2, and Zfatf4; and (3) C4-Zfatf1, Zfatf2, and Zfatf5. They profiled the bulk RNA-Seq data of (1) MEF cells, (2) C2 and SKM overexpressed induced iPS (C2+SKM iPS), (3) C2 and SKM overexpressed MEFs between 18 and 27 days (C2 + SKM early iPS), (4) C3 and SKM overexpressed induced iPS (C3 + SKM iPS), (5) C3 and SKM overexpressed MEFs between 18 and 27 days (C3 + SKM early iPS), (6) C4 and SKM overexpressed induced iPS (C4 + SKM iPS), (7) C4 and SKM overexpressed MEFs between 18 and 27 days (C4 + SKM early iPS), (8) SKM overexpressed MEFs (Empty SKM MEFs), (9) Oct4 and SKM overexpressed MEFs between 18 and 27 days (OSKM early iPS), and (10) Oct4 and SKM overexpressed iPS (OSKM iPS).

We took the data from MEFs as the control and the data from the genetically manipulated cells as the case. We ran CTSFinder and identified the significantly up-regulated gene clusters in each genetically manipulated cell (see “Permutation-Based Fold Change Test” in “Materials and Methods” section). We found that only gene cluster 1–5 was significantly up-regulated in C2 + SKM iPS, C3 + SKM iPS, C4 + SKM iPS, OSKM early iPS, and OSKM iPS (Figure 10B). We inferred gene cluster 1–5 to be signatures of stem/progenitor cells. This suggested that stem/progenitor cells appeared and expanded in these genetically manipulated cells.

Eguchi et al. (2016) clustered the above 10 cell types with fibroblast and pluripotency markers. The C2 + SKM iPS, C3 + SKM iPS, C4 + SKM iPS, and OSKM iPS with significant pluripotency marker expression were in the first group, and the other cell types were in the second group. However, the OSKM early iPS had a higher expression of pluripotency markers and a lower expression of fibroblast markers, compared to the other cells in the second group, suggesting that it occupied the transition point between fibroblast and pluripotency cells. The CTS gene clusters helped distinguish the stages of the induced iPSs.

Overall, the results demonstrated that the CTS gene clusters facilitated the identification of specific cell types between *in vitro*–cultured cells with either chemical or genetic manipulation from bulk RNA-Seq data.

### Identification of Specific Cell Types in the *in vivo* and *in vitro* Developing Mouse Retina

We tested the performance of CTS gene clusters on time-series bulk RNA-Seq data from developing mouse retina and developing mouse retina organoids derived from iPS cells to reveal the dynamics of cell types in the two development systems. Brooks et al. (2019) performed bulk RNA-Seq on developing and mature retina from 12 stages comprising four embryonic time points (E11, E12, E14, and E16) and eight postnatal time points (P0, P2, P4, P6, P10, P14, P21, and P28). They also performed bulk RNA-Seq on developing mouse retina organoids derived from iPS cells at 10 time points during differentiation (D0, D4, D7, D10, D12, D15, D18, D22, D25, and D32).

**FIGURE 11:**
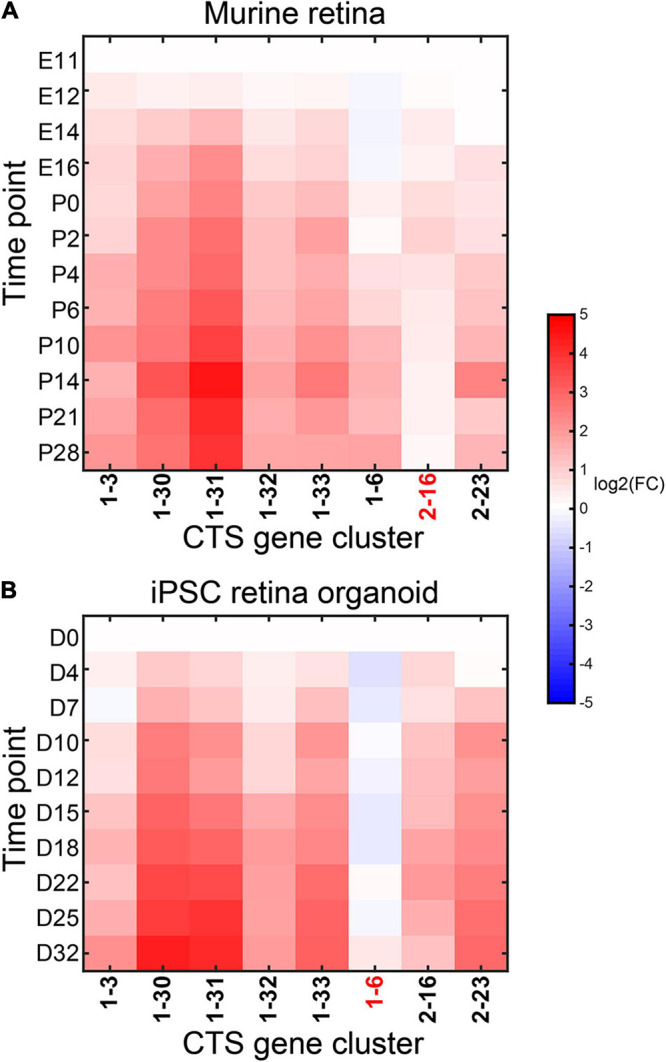
Expression fold change of the significantly up-regulated gene clusters in the *in vivo* **(A)** and *in vitro* **(B)** developing mouse retina.

We took the data from embryonic time point E11 as the control and the other data in the developing mouse retina cases. We took the data from D0 as the control and the other data cases in the developing mouse retina organoids. We ran CTSFinder and identified the significantly up-regulated gene clusters for each time point (see “Permutation-Based Fold Change Test” in “Materials and Methods” section). In the developing mouse retina, gene clusters 1–3, 1–30, 1–31, 1–32, 1–33, 1–6, and 2–23 were significantly up-regulated in at least one time point (Figure 11A). In the developing mouse retina organoids, gene clusters 1–3, 1–30, 1–31, 1–32, 1–33, 2–16, and 2–23 were significantly up-regulated in at least one time point (Figure 11B). The E types of 1–30, 1–31, and 1–33 include neurons, neuronal stem cells, oligodendrocyte precursor cells, astrocytes, and Bergmann glial cells (Supplementary Table 4). The three clusters were up-regulated during the development processes in both systems, indicating the development track of these cells. The E type of gene cluster 1–3 is ciliated columnar cells of tracheobronchial tree (Supplementary Table 4). Genes of 1–3 took part in the “cilium movement” and “cilium assembly” terms (Supplementary Table 6). Cluster 1–3 may share signature with a cell type with cilium in mouse retina, such as photoreceptor cilium, and indicate the cell type development in both systems. The E types of gene cluster 1–32 are endocrine cells in the pancreas (Supplementary Table 4). The GO term result showed that genes of 1–32 participated in the functions related to insulin (Supplementary Table 6). The gene cluster indicated a cell type similar to the endocrine cells in mouse retina. However, further investigation is needed. The E types of gene cluster 2–23 contain smooth muscle cells. The gene cluster indicated the development track of smooth muscle cells in both systems. Gene cluster 1–6 was only up-regulated in the developing mouse retina, and gene cluster 2–16 was specifically up-regulated in the developing mouse retina organoids. The E types of 1–6 are ventricular myocytes and atrial myocytes, and the E types of 2–16 include precursor B cells, early pro–B cells, and late pro–B cells. It is interesting to see that the gene clusters associated with different cell types were up-regulated in the development systems, respectively, which shed light on the distinct developmental fate of the *in vivo* and *in vitro* developing mouse retina. However, further wet laboratory investigations are needed to support the conclusion.

We used CIBERSORTx to estimate cell fractions in the *in vivo* and *in vitro* developing mouse retina bulk RNA-Seq data and compared the cell fractions between different time points (see “Application of CIBERSORTx to Estimate Cell Fractions in Bulk Samples” in “Materials and Methods” section). We identified the cell types with fold change > 2 at any time point and listed them in Supplementary Figure 3. The results revealed that neuronal stem cells were first expanded, and then neurons and astrocytes were expanded in both *in vivo* and *in vitro* developing mouse retina. However, the results from CIBERSORTx revealed that many cell types with small cell fractions were specifically expanded in each development system. They needed to be further investigated.

The CTSFinder captured the expansion of neurons, neuronal stem cells, and astrocytes in the two development systems. But it provided ambiguous results. CIBERSORTx specified the dynamics of these cell types. However, besides the cell types revealed from CIBERSORTx, CTSFinder identified the expansion of other cell types in both development systems, providing a more comprehensive view of the shared development track in the two systems.

## Discussion

We have identified 46 CTS gene clusters related to 83 mouse cell types using scRNA-Seq data from the Tabula Muris Senis project. We validated the CTS gene clusters in independent scRNA-Seq datasets. GO term enrichment analysis of the CTS gene sets revealed the specific functions of the gene set–associated cell types. Interestingly, we found some GO terms, such as “immune system process,” were enriched in a group of CTS gene clusters with distinct expression patterns, suggesting that the indicated functions were attributable to different genes in different cells. The multiple CTS gene clusters associated with the same cell type uncovered the potential functional similarity between different cell types. For example, hepatocytes and epithelial cells of proximal tubule tissue shared CTS gene cluster 2–2, enriched in the GO terms of “fatty acid metabolic process” and “ion transport” (Supplementary Table 6). Hepatocytes also shared CTS gene cluster 2–10 with Kupffer cells. The genes were enriched in the GO terms of “acute-phase response” and “blood coagulation” (Supplementary Table 6). The shared CTS genes suggested a functional similarity between hepatocytes and the two cell types. We then used CTS gene clusters and their E-types profile to identify different cell types between simulated bulk samples, between organs, between different development stages, between various *in vitro* culture conditions, and between *in vivo* and *in vitro* development systems. This demonstrated that the CTS gene clusters could be used for specific cell type identification between bulk samples.

Transcription factors (TFs) regulate cell division, cell growth, cell death throughout life, and cell migration and organization during embryonic development. We obtained 827 mouse TFs from TRRUST(v2) database (Han H. et al., 2018). We found 179 TFs in 36 CTS gene clusters (Supplementary Table 8). We obtained 881 mouse surface membrane proteins (SPs) from the Cell Surface Protein Atlas (Bausch-Fluck et al., 2015). We found 309 SPs in 38 CTS gene clusters (Supplementary Table 8). These genes can help us sort the special cell types and study their functions.

*In vitro* differentiation and expansion of stem and progenitor cells are widely applied to understand molecular mechanisms of cell differentiation and self-renewal. However, the microenvironment of *in vivo* cells and *in vitro* cells is significantly different. The cell identity of the cultured stem and progenitor cells, especially those after long-time culturing, needs to be clarified before drawing any conclusions when studying cell differentiation and expansion. Morphology, immunohistochemistry, and flow cytometry have all been applied to determining the cell identity of culture cells. However, the cultured cells may be differentiated into multiple cell types and highly heterogeneous. A comprehensive screen of all the possible cell types existing in the culture pool is required. In this respect, the RNA-Seq–based whole-genome screen potentially outperforms other methods.

We used genes specifically expressed in one or more cell types as CTS genes and found 46 CTS gene clusters for 83 cell types (Supplementary Table 4). The existing methods, including CTSFinder, rely heavily on information about CTS genes to identify cell types in bulk samples. A single-cell expression reference from bulk samples is prerequired for CIBERSORTx, Bisque, MuSiC, and some other methods to estimate the numerical proportions of the cell types in each bulk sample. CTen collected CTS genes mainly for mouse immune cells, and ssGSEA did not provide CTS genes for mouse cell types. The method with CTS genes covering more cell types will have more extensive applications. To our knowledge, the Tabula Muris Senis project provides the most comprehensive and high-quality scRNA-Seq data for mouse cell types. Thus, the identified 46 CTS gene clusters for 83 mouse cell types make CTSFinder unique and valuable.

The CTS gene clusters and the associated cell types (E types) were not one-to-one matched. This strategy could help us find CTS gene clusters for more cell types and extend CTS genes associated with a cell type, compared to the strategy of using genes specifically expressed in a unique cell type as CTS genes, such as CIBERSORTx and xCell adopted. However, multiple candidate cell types were reported, which led to ambiguous results in some conditions. Knowledge about the cell types that possibly appeared in the study will help us identify the specific cell types from the CTS gene cluster E-type list. However, we failed to specify the dynamics of relevant cell types in some circumstances. The CTS genes and cell types are one-to-one matched in CIBERSORTx and not one-to-one matched in CTSFinder, making CIBERSORTx outperform CTSFinder under this circumstance. The comparison between CTSFinder and CIBERSORTx in bulk RNA-Seq data from developing mouse liver, cultured giNPCs, and *in vivo* and *in vitro* developing mouse retina demonstrated it. People need to assess the benefits and risks before using CTSFinder.

The number of covered cell types is still limited. The Tabula Muris Senis project profiled 148 cell types in 20 or more cells using the SMART-Seq2 and 10x Genomics platforms. The CTS genes inferred from different data sources cannot be combined as a CTS gene set if they have not been evaluated across the data sources. The two platforms detect gene expression in different ways. SMART-Seq2 sequences mRNA in full length and detects gene expression with higher sensitivity, whereas 10x Genomics sequences mRNA in UTR region and provides higher throughput regarding cells. We did not merge the scRNA-Seq data from the two platforms because we could not normalize the noise coupling with the techniques. The multiple data sources can be merged to estimate CTS genes if they are well normalized. One hundred one cell types were analyzed here, and 83 were found with CTS gene clusters. However, the CTS gene clusters were determined by comparing cell types over the whole body. We might find CTS gene clusters for the failed cell types and extend the gene list of the existing CTS gene sets if we focused on, and compared, the cell types in a specific organ or organ system.

CTSFinder provides qualitative results. It can identify the cell type whose proportion in the bulk sample is significantly changed between two conditions. It does not specify the numerical proportions of the cell type in the two conditions. CIBERSORTx, Bisque, MuSiC, and some other methods provide quantitative solutions. They can infer the numerical proportions of the cell type in the bulk sample if an accurate single-cell expression reference is available. The Tabula Muris Senis project provides a comprehensive mouse single-cell expression reference. These quantitative solutions have not been evaluated with a single-cell expression reference of many cell types irrelevant to the studied bulk samples. Our application of CIBERSORTx with the single-cell expression reference from the Tabula Muris Senis project showed that many cell types with small cell fractions were reported, including the ones irrelevant to the studied bulk samples. People need to be cautious about using a comprehensive single-cell expression reference in these methods. For researchers with a single-cell expression reference for the bulk samples, these quantitative solutions are a better choice. However, CTSFinder will be attractive to those researchers who lack such a single-cell expression reference.

## Materials and Methods

### Data

We downloaded scRNA-Seq data using the SMART-Seq2 platform and 10x Genomics platform generated by the Tabula Muris Senis project from the GEO database (Clough and Barrett, 2016). For the SMART-Seq2 data, we removed cells with fewer than 5,000 counts and 500 detected genes. For the 10x Genomics data, we removed cells with fewer than 2,500 unique molecular identifiers and 500 detected genes. We also downloaded the cell annotation files for the cells sequenced by the two platforms. The bulk RNA-Seq data for 17 organs were downloaded from the GEO database, including five or six replicates per organ. The organ annotation file for the samples was also obtained. The mouse liver development bulk RNA-Seq data were downloaded from the GEO database, including Renaud et al. and Gong et al. Concerning Renaud et al.’s data, we downloaded gene expression profiles of samples at E17.5 and D0, D1, D3, D5, D10, D15, D20, D25, D30, D45, and D60, including three replicates per time point. In terms of Gong et al.’s data, we downloaded gene expression profiles of samples at E17.5 and E18.5; D1, D3, and D5; and W1, W2, W3, W6, and W8, including three replicates per time point. The mouse giNPCs bulk RNA-Seq data were downloaded from the GEO database, including samples at D1, D4, D7, D10, D14, D17, and D21 with duplicate samples per time point. The iPS cells bulk RNA-Seq data were downloaded from the GEO database, including 10 different conditions and three or four replicates per condition. The bulk RNA-Seq data for the *in vivo* and *in vitro* developing mouse retina systems were downloaded from the GEO database, including two or three replicates per time point. Detailed information concerning these data can be found in Supplementary Table 9.

### Calculation of Gene Expression Profiles of Cell Types

For the selected cell types, we calculated the gene expression profiles as follows. First, for each gene and cell type, the number of cells expressing the gene in the cell type was counted, and the percentage of cells in the cell type that express each gene was calculated. Second, the calculated percentages were taken as the expression level of the gene in the cell type. Finally, the expression levels for all genes in each of the cell types were obtained via this method.

### Gene Set Enrichment Analysis

Gene set enrichment analysis was conducted on the gene clusters using DAVID 6.8 (Huang da et al., 2009). In GO term enrichment analysis, terms from the “GOTERM_BP_DIRECT” ontology, which had Bonferroni-corrected *p* < 0.05, were taken as the significant terms (The Gene Ontology Consortium, 2019). In KEGG pathway enrichment analysis, the pathways with Bonferroni-corrected *p* < 0.05 were taken as the significant pathways (Kanehisa and Goto, 2000).

### Construction of the Simulated Datasets

We used the scRNA-Seq data sequenced by the SMART-Seq2 platform in 3-months-old mice and filtered cells as described in the “Data” section. We randomly selected one cell from each of the 101 cell types. Then we normalized the sequencing depth of each cell to 10,000 and scaled the read count of each gene accordingly. Next, we merged the 101 cells and summed the reads for each gene to yield a simulated bulk RNA-Seq dataset of cells from the 101 cell types. We repeated the processes three times to get three simulated bulk RNA-Seq datasets.

For each of the 101 cell types, we randomly selected 20 cells, normalized the sequencing depth to 10,000, scaled the read count of each gene, and merged 20 cells to get a simulated bulk RNA-Seq dataset for the cell type. We repeated the process three times to get three simulated bulk RNA-Seq datasets for each cell type.

### Permutation-Based Fold Change Test

Here, we describe a simple method named CTSFinder, which can identify the different cell types between case and control samples.

At first, we conducted differential gene expression analysis between the case and control samples. In the simulated bulk RNA-Seq data, we input the processed read files to DESeq2 (Love et al., 2014) and set the mode as “moderated log2 fold changes” to calculate the log2-transformed fold-change (*log*⁡2(*FC*)) value of each gene between samples. We downloaded raw read files pertaining to bulk RNA-Seq data from 17 organs and then used DESeq2 (Love et al., 2014), setting the mode as “moderated log2 fold changes” to calculate the log2-transformed fold-change (*log*⁡2(*FC*)) value of each gene between samples. In the bulk RNA-Seq data for the *in vivo* and *in vitro* developing mouse retina, we downloaded the CPM (counts per million reads mapped) value. In the other bulk RNA-Seq data, we downloaded the FPKM (fragments per kilobase of exon model per million reads mapped) value. We calculated its median values in the case samples and the control samples for each gene. Then, for each gene, we selected the large one between 1 and its median value in the case samples and the large one between 1 and its median value in the control samples and calculated the log2-transformed fold-change (*log*⁡2(*FC*)) value with the two values.

Then, we filtered out the genes with *log*⁡2(*FC*) equal to zero. We counted the sequenced genes in each of the 46 CTS gene clusters and selected those clusters with 10 or more expressed genes to conduct further analysis.

Third, for a gene cluster, we calculated the median of *log*⁡2(*FC*)value of its genes as median(*log*⁡2(*FC*)_all_). Then, we shuffled the *log*⁡2(*FC*) value of all expressed genes 10,000 times and calculated the median(*log*⁡2(*FC*)_all_) of the gene cluster as the median(log2(*FC*)_perm_) at each time to obtain a median(log2(*FC*)_perm_) set. Next, we calculated the frequency of the value in median(log2(*FC*)_perm_)set equal to or higher than median(log2(*FC*)_all_) as *p* value if median(log2(*FC*)_all_)≥0. We calculated the frequency of the value in the median(log2(*FC*)_perm_)set equal to or lower than median(log2(*FC*)_all_) as *p* value if median(log2(*FC*)_all_) < 0. We calculated median(log2(*FC*)_all_) and *p* value for each gene cluster in this way.

Finally, we identified the significant gene clusters with median(log2(*FC*)_all_) and *p* value. We identified the significantly up-regulated gene clusters in bulk simulated RNA-Seq data and bulk organ RNA-Seq data with median(log2(*FC*)_all_) > 1 and *p* < 0.001. We identified the significantly up- or down-regulated gene clusters in the mouse developing liver RNA-Seq data with median(log2(*FC*)_all_) > 1ormedian(log2(*FC*)_all_) < −1 and *p* < 0.001. We identified the significantly up-regulated gene clusters in giNPC data and iPS cell data with median(log2(*FC*)_all_) > 1 and *p* < 0.001. We identified the significantly up-regulated gene clusters in the *in vivo* and *in vitro* developing mouse retina data with median(log2(*FC*)_all_) > 1 and *p* < 0.001.

### Application of CIBERSORTx to Estimate Cell Fractions in Bulk Samples

We used the CIBERSORTx toolkit^1^ to estimate cell fractions in the different time points of developing mouse livers, *in vitro*–cultured giNPCs, and *in vivo* and *in vitro* developing mouse retina. The scRNA-Seq data from 3-months-old mice sequenced by the SMART-Seq2 platform from the Tabula Muris Senis project were taken as a scRNA-Seq reference. We input read count matrix of the scRNA-Seq data into the toolkit to get a signature matrix. The parameters are listed in Supplementary Table 10. We input the signature matrix and each bulk RNA-Seq dataset to estimate cell fractions using the CIBERSORTx-B model. The parameters are also listed in Supplementary Table 10. In the bulk RNA-Seq data for the *in vivo* and *in vitro* developing mouse retina, CPM values were used; in the other data, FPKM values were used.

We then compared the cell fractions between the start time point and other time points in each bulk RNA-Seq dataset. E17.5 was set as the start time point in the developing mouse livers data; D1 was taken as the start time point in the *in vitro*–cultured giNPC data; E11 and D0 were set as the start time points in the *in vivo* and *in vitro* developing mouse retina data, respectively. In each bulk RNA-Seq dataset, we calculated the fold changes of cell fractions at the other time points with respect to that at the start time point for a cell type: at first, cell fractions small than 0.01 were input with 0.01; then, cell fractions of samples from the same time point were averaged; finally, fold changes of cell fractions between the other time points and the start time point were calculated. We calculated the fold changes for all 101 cell types in each dataset via this method.

## Data Availability Statement

Publicly available datasets were analyzed in this study. This data can be found here: All the data are publicly available from the GEO database [https://www.ncbi.nlm.nih.gov/geo/]. The single-cell RNA-Seq data and coupling cell annotation file can be found under accession code GSE149590; the bulk RNA-Seq data of 17 tissues and organs and associated sample information file can be found with accession code GSE132040; the liver tissue RNA-Seq data can be found under accession code GSE58827 and GSE132034; the *in vitro* cultured giNPCs and iPS cells data can be found with accession code GSE76857 and GSE89219; and the *in vivo* and *in vitro* developing mouse retina data can be found with accession code GSE101986 and GSE102794.

## Author Contributions

XH and CW contributed to the conception and design of the study. CW organized the database and performed the statistical analysis. XH, LL, BC, and CW drafted the manuscript. All authors contributed to manuscript revision, read, and approved the submitted version.

## Conflict of Interest

The authors declare that the research was conducted in the absence of any commercial or financial relationships that could be construed as a potential conflict of interest.

## Footnotes

1 https://cibersortx.stanford.edu/

## Supplementary Material

Supplementary Figure 1 Cell fractions of 101 cell types in bulk samples from developing mouse livers. Cell fractions of 101 cell types in the bulk samples from Renaud et al. data **(A)** and Gong et al. data **(B)** were estimated by CIBERSORTx. The samples from different time points are indicated. The names of cell types with fold change > 2 or fold change < 0.5 are listed in the figures, and the names of all cell types are listed in Supplementary Table 1.

Supplementary Figure 2 Cell fractions of 101 cell types in bulk samples from cultured giNPCs. The samples from different time points are indicated. The names of cell types with fold change > 2 are listed in the figure, and the names of all cell types are listed in Supplementary Table 1.

Supplementary Figure 3 Cell fractions of 101 cell types in bulk samples from *in vivo* and *in vitro* developing mouse retina. Cell fractions of 101 cell types in the bulk samples from *in vivo* **(A)** and *in vitro* **(B)** developing mouse retina were estimated by CIBERSORTx. The samples from different time points are indicated. The names of cell types with fold change > 2 are listed in the figures, and the names of all cell types are listed in Supplementary Table 1.

## References

[B1] Aran D. Hu Z. Butte A. J. (2017). xCell: digitally portraying the tissue cellular heterogeneity landscape. *Genome Biol.* 18:220. 10.1186/s13059-017-1349-1 29141660 PMC5688663

[B2] Barbie D. A. Tamayo P. Boehm J. S. Kim S. Y. Moody S. E. Dunn I. F. (2009). Systematic RNA interference reveals that oncogenic KRAS-driven cancers require TBK1. *Nature* 462 108–112. 10.1038/nature08460 19847166 PMC2783335

[B3] Bausch-Fluck D. Hofmann A. Bock T. Frei A. P. Cerciello F. Jacobs A. (2015). A mass spectrometric-derived cell surface protein atlas. *PLoS One* 10:e0121314. 10.1371/journal.pone.0121314 25894527 PMC4404347

[B4] Brooks M. J. Chen H. Y. Kelley R. A. Mondal A. K. Nagashima K. De Val N. (2019). Improved retinal organoid differentiation by modulating signaling pathways revealed by comparative transcriptome analyses with development in vivo. *Stem Cell Rep.* 13 891–905. 10.1016/j.stemcr.2019.09.009 31631019 PMC6895716

[B5] Clough E. Barrett T. (2016). The gene expression omnibus database. *Methods Mol. Biol.* 1418 93–110. 10.1007/978-1-4939-3578-9_5 27008011 PMC4944384

[B6] Deng Y. Bao F. Dai Q. Wu L. F. Altschuler S. J. (2019). Scalable analysis of cell-type composition from single-cell transcriptomics using deep recurrent learning. *Nat. Methods* 16 311–314. 10.1038/s41592-019-0353-7 30886411 PMC6774994

[B7] Eguchi A. Wleklinski M. J. Spurgat M. C. Heiderscheit E. A. Kropornicka A. S. Vu C. K. (2016). Reprogramming cell fate with a genome-scale library of artificial transcription factors. *Proc. Natl. Acad. Sci. U.S.A.* 113 E8257–E8266. 10.1073/pnas.1611142114 27930301 PMC5187731

[B8] Franzén O. Gan L. M. Björkegren J. L. M. (2019). PanglaoDB: a web server for exploration of mouse and human single-cell RNA sequencing data. *Database (Oxford)* 2019:baz046. 10.1093/database/baz046 30951143 PMC6450036

[B9] Frishberg A. Peshes-Yaloz N. Cohn O. Rosentul D. Steuerman Y. Valadarsky L. (2019). Cell composition analysis of bulk genomics using single-cell data. *Nat. Methods* 16 327–332. 10.1038/s41592-019-0355-5 30886410 PMC6443043

[B10] Gao R. Xiu W. Zhang L. Zang R. Yang L. Wang C. (2017). Direct induction of neural progenitor cells transiently passes through a partially reprogrammed state. *Biomaterials* 119 53–67. 10.1016/j.biomaterials.2016.12.007 28006658

[B11] Gong T. Zhang C. Ni X. Li X. Li J. Liu M. (2020). A time-resolved multi-omic atlas of the developing mouse liver. *Genome Res.* 30 263–275. 10.1101/gr.253328.119 32051188 PMC7050524

[B12] Han H. Cho J. W. Lee S. Yun A. Kim H. Bae D. (2018). TRRUST v2: an expanded reference database of human and mouse transcriptional regulatory interactions. *Nucleic Acids Res.* 46 D380–D386. 10.1093/nar/gkx1013 29087512 PMC5753191

[B13] Han X. Wang R. Zhou Y. Fei L. Sun H. Lai S. (2018). Mapping the mouse cell atlas by microwell-seq. *Cell* 172 1091–1107.e1017. 10.1016/j.cell.2018.02.001 29474909

[B14] Han X. Zhou Z. Fei L. Sun H. Wang R. Chen Y. (2020). Construction of a human cell landscape at single-cell level. *Nature* 581 303–309. 10.1038/s41586-020-2157-4 32214235

[B15] Hatano A. Chiba H. Moesa H. A. Taniguchi T. Nagaie S. Yamanegi K. (2011). CELLPEDIA: a repository for human cell information for cell studies and differentiation analyses. *Database (Oxford)* 2011:bar046. 10.1093/database/bar046 22039163 PMC3204613

[B16] Hu P. Zhang W. Xin H. Deng G. (2016). Single cell isolation and analysis. *Front. Cell Dev. Biol.* 4:116. 10.3389/fcell.2016.00116 27826548 PMC5078503

[B17] Huang da W. Sherman B. T. Lempicki R. A. (2009). Systematic and integrative analysis of large gene lists using DAVID bioinformatics resources. *Nat. Protoc.* 4 44–57. 10.1038/nprot.2008.211 19131956

[B18] Jew B. Alvarez M. Rahmani E. Miao Z. Ko A. Garske K. M. (2020). Accurate estimation of cell composition in bulk expression through robust integration of single-cell information. *Nat. Commun.* 11:1971. 10.1038/s41467-020-15816-6 32332754 PMC7181686

[B19] Kanehisa M. Goto S. (2000). KEGG: kyoto encyclopedia of genes and genomes. *Nucleic Acids Res.* 28 27–30. 10.1093/nar/28.1.27 10592173 PMC102409

[B20] Kassambara A. Mundt F. (2019). *factoextra: Extract and Visualize the Results of Multivariate Data Analyses. R Package Version 1.0.6.* Available online at: https://CRAN.Rproject.org/package=factoextra

[B21] Le T. Aronow R. A. Kirshtein A. Shahriyari L. (2020). A review of digital cytometry methods: estimating the relative abundance of cell types in a bulk of cells. *Brief. Bioinform.* 1:bbaa219. 10.1093/bib/bbaa219 33003193 PMC8293826

[B22] Love M. I. Huber W. Anders S. (2014). Moderated estimation of fold change and dispersion for RNA-seq data with DESeq2. *Genome Biol.* 15:550. 10.1186/s13059-014-0550-8 PMC4302049 25516281

[B23] Newman A. M. Steen C. B. Liu C. L. Gentles A. J. Chaudhuri A. A. Scherer F. (2019). Determining cell type abundance and expression from bulk tissues with digital cytometry. *Nat. Biotechnol.* 37 773–782. 10.1038/s41587-019-0114-2 31061481 PMC6610714

[B24] Panina Y. Karagiannis P. Kurtz A. Stacey G. N. Fujibuchi W. (2020). Human Cell Atlas and cell-type authentication for regenerative medicine. *Exp. Mol. Med.* 52 1443–1451. 10.1038/s12276-020-0421-1 32929224 PMC8080834

[B25] Polanco A. Kuang B. Yoon S. (2020). Bioprocess technologies that preserve the quality of iPSCs. *Trends Biotechnol.* 38 1128–1140. 10.1016/j.tibtech.2020.03.006 32941792

[B26] Renaud H. J. Cui Y. J. Lu H. Zhong X. B. Klaassen C. D. (2014). Ontogeny of hepatic energy metabolism genes in mice as revealed by RNA-sequencing. *PLoS One* 9:e104560. 10.1371/journal.pone.0104560 25102070 PMC4125194

[B27] Schachtrup C. Le Moan N. Passino M. A. Akassoglou K. (2011). Hepatic stellate cells and astrocytes: Stars of scar formation and tissue repair. *Cell Cycle* 10 1764–1771. 10.4161/cc.10.11.15828 21555919 PMC3142460

[B28] Shibamiya A. Schulze E. Krauss D. Augustin C. Reinsch M. Schulze M. L. (2020). Cell banking of hiPSCs: a practical guide to cryopreservation and quality control in basic research. *Curr. Protoc. Stem Cell Biol.* 55:e127. 10.1002/cpsc.127 32956561

[B29] Shoemaker J. E. Lopes T. J. Ghosh S. Matsuoka Y. Kawaoka Y. Kitano H. (2012). CTen: a web-based platform for identifying enriched cell types from heterogeneous microarray data. *BMC Genomics* 13:460. 10.1186/1471-2164-13-460 22953731 PMC3473317

[B30] Stachelscheid H. Seltmann S. Lekschas F. Fontaine J. F. Mah N. Neves M. (2014). CellFinder: a cell data repository. *Nucleic Acids Res.* 42 D950–D958. 24304896 10.1093/nar/gkt1264 PMC3965082

[B31] Sun K. Jiang P. Chan K. C. Wong J. Cheng Y. K. Liang R. H. (2015). Plasma DNA tissue mapping by genome-wide methylation sequencing for noninvasive prenatal, cancer, and transplantation assessments. *Proc. Natl. Acad. Sci. U.S.A.* 112 E5503–E5512. 26392541 10.1073/pnas.1508736112 PMC4603482

[B32] Tabula Muris Consortium, Overall coordination, Logistical coordination, Organ collection and processing, Library preparation and sequencing, Computational data analysis (2018). Single-cell transcriptomics of 20 mouse organs creates a Tabula Muris. *Nature* 562 367–372. 10.1038/s41586-018-0590-4 30283141 PMC6642641

[B33] Tabula Muris Consortium. (2020). A single-cell transcriptomic atlas characterizes ageing tissues in the mouse. *Nature* 583 590–595. 10.1038/s41586-020-2496-1 32669714 PMC8240505

[B34] Tang F. Barbacioru C. Wang Y. Nordman E. Lee C. Xu N. (2009). mRNA-Seq whole-transcriptome analysis of a single cell. *Nat. Methods* 6 377–382. 10.1038/nmeth.1315 19349980

[B35] The Gene Ontology Consortium (2019). The gene ontology resource: 20 years and still GOing strong. *Nucleic Acids Res.* 47 D330–D338. 30395331 10.1093/nar/gky1055 PMC6323945

[B36] Tsoucas D. Dong R. Chen H. Zhu Q. Guo G. Yuan G. C. (2019). Accurate estimation of cell-type composition from gene expression data. *Nat. Commun.* 10:2975. 10.1038/s41467-019-10802-z PMC6611906 31278265

[B37] Vrba L. Futscher B. W. (2018). A suite of DNA methylation markers that can detect most common human cancers. *Epigenetics* 13 61–72. 10.1080/15592294.2017.1412907 29212414 PMC5836970

[B38] Wang X. Park J. Susztak K. Zhang N. R. Li M. (2019). Bulk tissue cell type deconvolution with multi-subject single-cell expression reference. *Nat. Commun.* 10:380. 10.1038/s41467-018-08023-x PMC6342984 30670690

[B39] Zhang X. Lan Y. Xu J. Quan F. Zhao E. Deng C. (2019). CellMarker: a manually curated resource of cell markers in human and mouse. *Nucleic Acids Res.* 47 D721–D728. 30289549 10.1093/nar/gky900 PMC6323899

