# Supplementary figures and images for: Using Cell Type–Specific Genes to Identify Cell-Type Transitions Between Different in vitro Culture Conditions
Source: Front Cell Dev Biol. 2021 Jun 25;9:644261. doi: 10.3389/fcell.2021.644261 (PMC8267371; doi:10.3389/fcell.2021.644261)

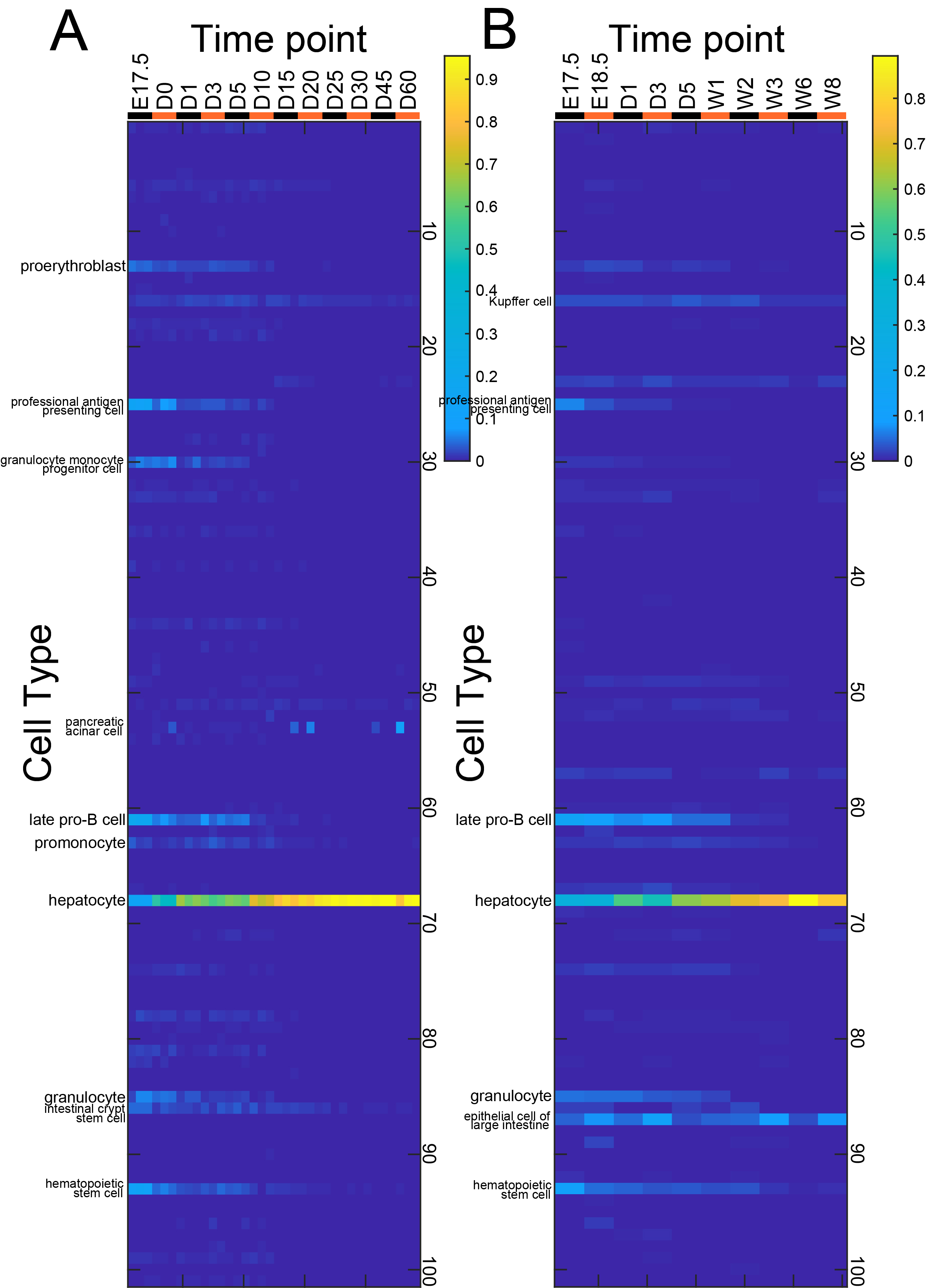

Supplement: Supplementary Figure 1 — Cell fractions of 101 cell types in bulk samples from developing mouse livers. Cell fractions of 101 cell types in the bulk samples from Renaud et al. data (A) and Gong et al. data (B) were estimated by CIBERSORTx. The samples from different time points are indicated. The names of cell types with fold change > 2 or fold change < 0.5 are listed in the figures, and the names of all cell types are listed in Supplementary Table 1. [file Image_1.JPEG]

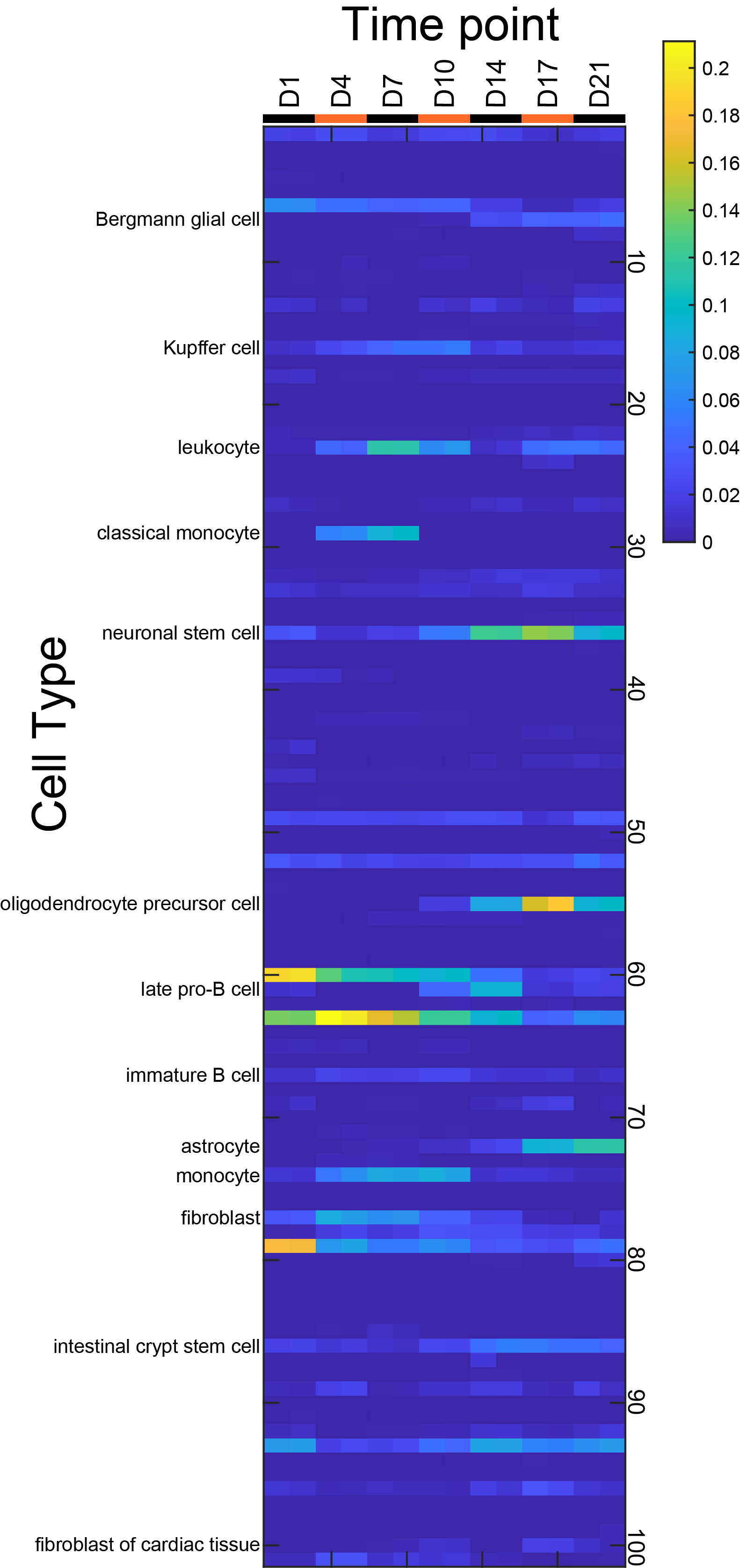

Supplement: Supplementary Figure 2 — Cell fractions of 101 cell types in bulk samples from cultured giNPCs. The samples from different time points are indicated. The names of cell types with fold change > 2 are listed in the figure, and the names of all cell types are listed in Supplementary Table 1. [file Image_2.JPEG]

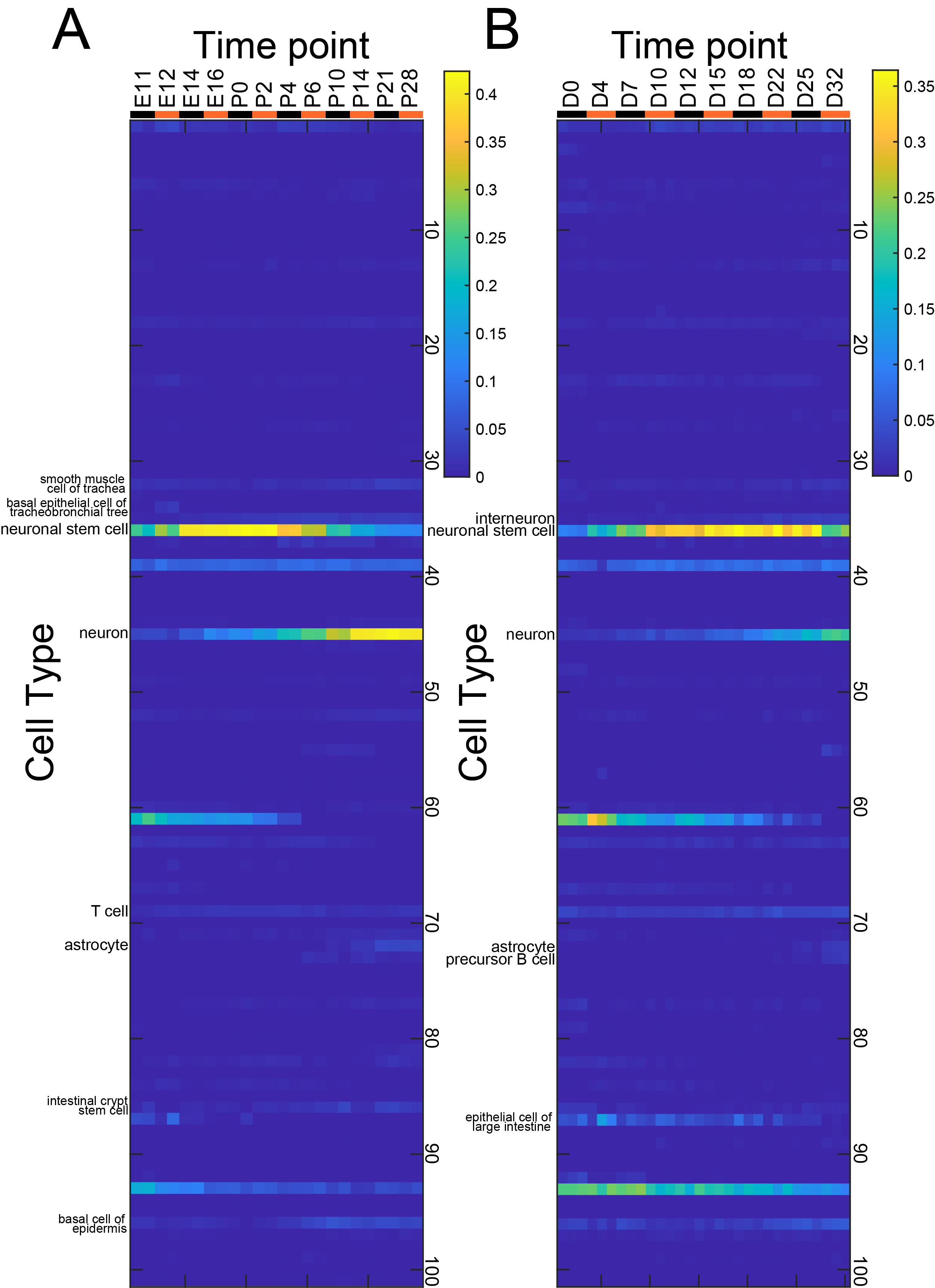

Supplement: Supplementary Figure 3 — Cell fractions of 101 cell types in bulk samples from in vivo and in vitro developing mouse retina. Cell fractions of 101 cell types in the bulk samples from in vivo (A) and in vitro (B) developing mouse retina were estimated by CIBERSORTx. The samples from different time points are indicated. The names of cell types with fold change > 2 are listed in the figures, and the names of all cell types are listed in Supplementary Table 1. [file Image_3.JPEG]
